# A Case of Rapidly Diagnosed Mycobacterium intracellulare in a Frail Geriatric Patient With Multimorbidity

**DOI:** 10.7759/cureus.62313

**Published:** 2024-06-13

**Authors:** Shengmin Yang, Zhengquan He, Ning Zhang, Minya Lu

**Affiliations:** 1 Key Laboratory of Endocrinology of National Health Commission, Department of Endocrinology, State Key Laboratory of Complex Severe and Rare Diseases, Peking Union Medical College Hospital, Peking Union Medical College, Chinese Academy of Medical Sciences, Beijing, CHN; 2 Department of Geriatrics, Peking Union Medical College Hospital, Peking Union Medical College, Chinese Academy of Medical Sciences, Beijing, CHN; 3 Department of Clinical Laboratory, Peking Union Medical College Hospital, Peking Union Medical College, Chinese Academy of Medical Sciences, Beijing, CHN

**Keywords:** collaborative care, multimorbidity, elderly, metagenomic next-generation sequencing, mycobacterium intracellulare

## Abstract

The prevalence of non-tuberculous mycobacteria (NTM) infections has been on the rise in recent years, especially among the elderly population and other immunocompromised groups. Risk factors for NTM infections include advanced age, preexisting pulmonary diseases, and low body mass index. This study presents a case of NTM pulmonary disease attributed to *Mycobacterium intracellulare*, which was rapidly identified using metagenomic next-generation sequencing (mNGS). An 82-year-old male presented with persistent fever, cough, and shortness of breath. Initial assessments revealed an elevated white blood cell count and high-sensitivity C-reactive protein, with chest CT showing newly formed nodular shadows and cavity formation. Sputum tests confirmed NTM infection through positive acid-fast staining and mNGS, which rapidly identified *M. intracellulare* within 48 hours. Subsequent sputum samples confirmed the diagnosis using traditional methods. The patient had a complex medical history, including pulmonary tuberculosis, chronic pancreatitis, chronic hepatitis B, diabetes, and malnutrition. The patient was treated with a combination of cefotaxime, moxifloxacin, clarithromycin, and acetylcysteine, in addition to receiving nutritional support. After the treatment, there was an improvement in symptoms, normalization of body temperature, and a decrease in cough and sputum production. This case highlights the significance of mNGS in promptly diagnosing and treating NTM pulmonary disease, especially in elderly patients with various underlying health conditions. The collaborative effort among different medical specialties enabled more thorough patient care, ultimately leading to better outcomes. Incorporating cutting-edge diagnostic techniques such as mNGS alongside a holistic treatment approach is crucial for the successful management of NTM infections in at-risk populations.

## Introduction

The past decade has seen an increase in the prevalence of non-tuberculous mycobacteria (NTM) infections globally, possibly due to a growing aging population and an increasing number of immunocompromised individuals, such as those undergoing organ transplantations and other procedures. Other risk factors include preexisting pulmonary diseases such as chronic obstructive pulmonary disease or cystic fibrosis, low body mass index (BMI), and the use of immunosuppressants [1]. NTM colonizes soil and water resources and is transmitted from the environment (especially water) to humans through the respiratory tract, gastrointestinal tract, and skin. The distribution of NTM species varies by region. In Beijing, the main source of NTM lung disease is *Mycobacterium intracellulare*, a key species within the *Mycobacterium avium* complex (MAC), responsible for 31.8% of cases [2].

Non-tuberculous mycobacterial pulmonary disease presents with diverse patterns on CT scans, often resembling other pulmonary diseases such as tuberculosis or bronchiectasis. Common CT findings in NTM pulmonary disease include the fibrocavitary pattern, the nodular-bronchiectatic pattern, and hypersensitivity pneumonitis [3,4]. Specifically, the fibrocavitary pattern is seen in patients with a history of smoking or preexisting lung diseases, typically indicating a more aggressive form of the disease [5]. Thin-walled cavities and fibrotic lesions are typically found in the upper lobes. The nodular-bronchiectatic pattern is characterized by bronchiectasis and the presence of small nodules, sometimes with a tree-in-bud appearance suggesting bronchiolar inflammation and infection [5,6]. Patchy areas of consolidation may be observed, particularly surrounding the bronchiectatic regions.

The diagnosis of NTM requires microbiological tests to confirm its presence and identify its subtype [7]. Conventional culture-based methods often struggle to detect *Mycobacterium* species due to their strict growth conditions and slow proliferation rates. Metagenomic next-generation sequencing (mNGS) is a high-throughput sequencing method that addresses these limitations by rapidly analyzing genetic material in clinical samples with high sensitivity and broad coverage [8]. Therefore, metagenomics may be particularly effective in urgent cases involving at-risk groups with positive acid-fast stains, where prompt action is necessary before microbiology cultures can identify potential pathogens to prevent further severe lung damage. In this case study, we present the findings of a frail elderly patient with multiple health conditions, whose *M. intracellulare* infection leading to lung cavity formation was identified within 48 hours using mNGS.

## Case presentation

An 82-year-old male patient was admitted to the Geriatric Department of Peking Union Medical College Hospital with a two-week history of persistent afternoon fever (maximum temperature 37.8-38.2°C), along with prolonged cough, grayish-purulent sputum, shortness of breath, and nonspecific chest tightness. Initial blood tests revealed a white blood cell count of 9.27 × 10^9^/L, with 75.1% neutrophils. Liver and kidney function tests showed normal results (alanine transaminase: 15 U/L, albumin: 35 g/L, creatinine: 63 μmol/L), as well as a normal procalcitonin level (0.12 ng/mL), but elevated high-sensitivity C-reactive protein (150.5 mg/L).

**Figure 1:**
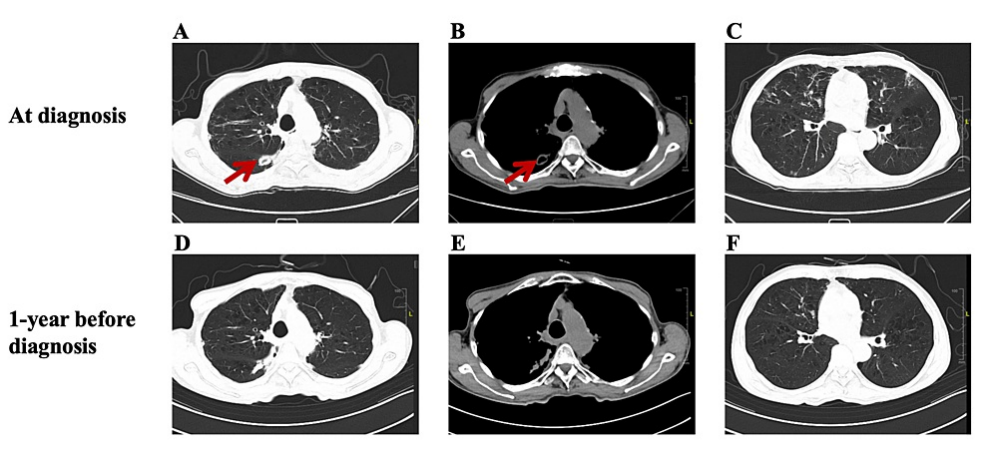
The patient’s lung lesions on non-contrast CT scans. A-C, thoracic CT at diagnosis: A. Lung cavity in the right lower lobe on the lung window. B. Lung cavity in the right lower lobe on the mediastinal window. C. Patchy cord-like shadows in both lower lobes on the lung window. All red arrows point to the lung cavity. D-F, thoracic CT performed one year before diagnosis: D. The corresponding CT scan slice to A. E. The corresponding CT scan slice to B. F. The corresponding CT scan slice to C.

**Figure 2:**
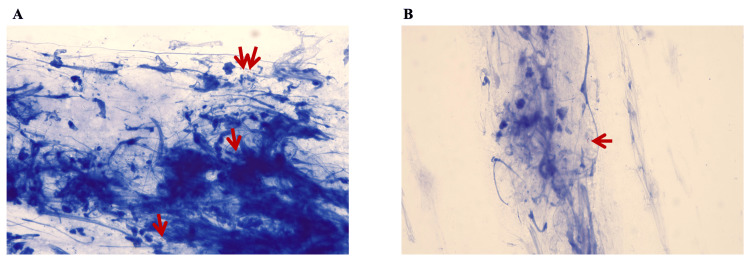
Acid-fast stain of the sputum. A. A view of the acid-fast stain. B. Another view of the acid-fast stain. All red arrows point to acid-fast bacilli.

Chest CT scans displayed nodular shadows in the right lower lobe along the bronchovascular bundles, nodules in the dorsal segment of the right lower lobe with adjacent pleural thickening and new cavity formation, and multiple patchy cord-like shadows in both lower lobes (Figure 1). Sputum tests confirmed acid-fast staining positivity in the first sample (Figure 2) and mycobacterial DNA positivity for NTM in two separate samples. mNGS identified the same pathogen within 48 hours, while traditional Sanger sequencing detected *M. intracellulare* in two other samples two to three days later. The *Mycobacterium tuberculosis*/rifampicin resistance test was negative for three sputum samples. Blood enzyme-linked immunospot assay for interferon-γ (T-SPOT.TB test kit: Oxford Immunotec, Oxford, United Kingdom) showed discordant results: 60FC/10S6MC spots after being stimulated by early secreted antigenic target-6 and 0 FC/10S6MC spots after incubating with culture filtrate protein-10, not supporting active tuberculosis. Various other tests for bacteria, fungi, *Nocardia*, India ink staining, pneumocystis pneumonia DNA, actinomycetes culture, and *Legionella pneumophila* antibody were unremarkable. Additionally, serum tumor markers (α-fetoprotein, carbohydrate antigen 19-9, carbohydrate antigen 24-2, carcinoembryonic antigen, squamous cell carcinoma antigen) were negative.

Since the onset of symptoms, the patient’s overall health, appetite, and sleep quality had been satisfactory, with dry stools two to three times a day and a weight loss of around 2 kg. The patient’s medical history included pulmonary tuberculosis which was properly treated 40 years ago, with sputum tests for tuberculosis remaining negative thereafter. Other past medical history included duodenal ulcer, chronic hepatitis B infection (with low HBV-DNA levels), syphilis, chronic pancreatitis (managed with pancreatic duct stenting twice), type 2 diabetes (controlled with glipizide, dapagliflozin, and acarbose), suspected glaucoma, and cataracts. Upon admission, the patient’s physical examination revealed stable vital signs (body temperature: 38.2°C, blood pressure: 137/70 mmHg, SpO_2_: 94% on room air, heart rate: 78 beats per minute, respiratory rate: 18 breaths per minute). The patient was alert, underweight, and malnourished (BMI: 16.53 kg/m^2^), with a regular heart rhythm and no significant heart murmurs. Coarse breath sounds with intermittent moist rales were detected in both lungs. The abdomen was soft without tenderness or rebound pain, and no leg swelling was observed. Geriatric assessment scores were as follows: Activities of Daily Living score of 6, Instrumental Activities of Daily Living score of 8, and Nutritional Risk Screening 2002 (NRS-2002) score of 5.

Based on these findings, a multidisciplinary team consisting of geriatricians, infectious disease specialists, and respiratory physicians diagnosed the patient with NTM pulmonary disease leading to cavitation. Treatment included cefotaxime 2 g intravenous infusion every 12 hours (switched to oral cefaclor 100 mg twice daily after one week of intravenous therapy and stopped after a total of two weeks of cephalosporin treatment), moxifloxacin 0.4 g daily, and clarithromycin 500 mg twice daily. Besides postural drainage, the patient received acetylcysteine aerosol inhalation, 3 mL twice daily, to aid in mucus clearance. Following treatment, the patient’s body temperature normalized, and cough and sputum production were reduced. The multidisciplinary team also included nutritionists who assessed the patient’s nutritional status, endocrinologists who adjusted the antidiabetic therapy, and pharmacists who evaluated whether there were any unwanted drug-drug interactions. A nutritional assessment revealed malnutrition (NRS-2002 score of 5). Initially, 1,000 mL of enteral nutritional suspension (diabetes) (TPF-DM) was administered, but the patient could only tolerate 500 mL. The dose of glipizide was adjusted to 30 mg daily orally to prevent hypoglycemia. Blood glucose levels were effectively managed, with fasting levels ranging between 5 and 7 mmol/L and postprandial levels between 11 and 13 mmol/L.

## Discussion

The rising occurrence of NTM infections, especially among the elderly populations with preexisting lung disease, poses a substantial challenge to public health worldwide. This instance highlights the crucial role of prompt and reliable diagnostic methods in the treatment of NTM pulmonary disease, particularly in older patients with multiple underlying health conditions. While traditional culture-based techniques are useful, they often struggle to detect NTM due to their strict growth demands and slow growth rates. This issue is particularly notable in species such as *M. intracellulare*, which is common in areas like Beijing and plays a significant role in NTM lung disease.

This case demonstrated common risk factors for NTM infection, including older age, a history of pulmonary diseases, low BMI, and multiple underlying health conditions (particularly chronic pancreatitis affecting the patient’s nutritional status). The clinical symptoms of persistent fever, cough, and cavitary lung lesions emphasized the aggressive nature of the disease in individuals with compromised health. CT scans revealed typical radiological patterns of NTM pulmonary disease, such as nodular shadows, pleural thickening, and formation of new cavities, which are crucial for distinguishing it from other pulmonary conditions such as bronchiectasis.

The use of mNGS was essential in this case, allowing for the rapid identification of *M. intracellulare* within 48 hours. Previous studies have shown that mNGS exhibited a sensitivity of 90.2% and specificity of 100% for NTM detection in 118 patients, significantly surpassing the performance of acid-fast staining (42.0%) and culture (77.0%) (p < 0.001) [9]. Moreover, when dealing with co-infection involving multiple NTM species, mNGS has the capability to simultaneously detect various NTM species [10]. Therefore, this high-throughput sequencing technique showed superior speed and sensitivity compared to traditional diagnostic methods, enabling timely and accurate diagnosis. The early detection facilitated the prompt initiation of targeted antibiotic therapy, including cefotaxime, moxifloxacin, and clarithromycin, alongside supportive measures such as acetylcysteine for expectoration relief and nutritional support to address malnutrition.

This case somewhat mimics Lady Windermere syndrome, where elderly women with low BMI have MAC pulmonary infections localized to the right middle lobe due to inadequate drainage of lung secretions [11]. Therefore, postural drainage and acetylcysteine aerosol inhalation were used to ensure smooth sputum drainage. Moreover, the multidisciplinary approach, which included specialists from geriatrics, infectious disease, respiratory medicine, endocrinology, nutrition, and pharmacy, played a crucial role in addressing the complex needs of the patient. This collaborative effort facilitated comprehensive care by addressing both the infection and the comorbidities, such as diabetes and malnutrition.

## Conclusions

This case underscores the importance of integrating advanced diagnostic tools such as mNGS into a multidisciplinary approach to improve treatment outcomes in individuals at risk of NTM pulmonary disease.

## References

[1] Dartois V, Dick T (2024). Therapeutic developments for tuberculosis and nontuberculous mycobacterial lung disease. Nat Rev Drug Discov.

[2] Huang JJ, Li YX, Zhao Y, Yang WH, Xiao M, Kudinha T, Xu YC (2020). Prevalence of nontuberculous mycobacteria in a tertiary hospital in Beijing, China, January 2013 to December 2018. BMC Microbiol.

[3] Pennington KM, Vu A, Challener D, Rivera CG, Shweta FN, Zeuli JD, Temesgen Z (2021). Approach to the diagnosis and treatment of non-tuberculous mycobacterial disease. J Clin Tuberc Other Mycobact Dis.

[4] Musaddaq B, Cleverley JR (2020). Diagnosis of non-tuberculous mycobacterial pulmonary disease (NTM-PD): modern challenges. Br J Radiol.

[5] Johnson MM, Odell JA (2014). Nontuberculous mycobacterial pulmonary infections. J Thorac Dis.

[6] Weiss CH, Glassroth J (2012). Pulmonary disease caused by nontuberculous mycobacteria. Expert Rev Respir Med.

[7] Kurz SG, Zha BS, Herman DD, Holt MR, Daley CL, Ruminjo JK, Thomson CC (2020). Summary for clinicians: 2020 clinical practice guideline summary for the treatment of nontuberculous mycobacterial pulmonary disease. Ann Am Thorac Soc.

[8] Chiu CY, Miller SA (2019). Clinical metagenomics. Nat Rev Genet.

[9] Zhang X, Chen H, Lin Y, Yang M, Zhao H, Hu J, Han D (2023). Diagnosis of non-tuberculous mycobacterial pulmonary disease by metagenomic next-generation sequencing on bronchoalveolar lavage fluid. Infect Drug Resist.

[10] Chen X, Zhu J, Liu Z, Ye J, Yang L, Zhang Z (2023). Mixed infection of three nontuberculous mycobacteria species identified by metagenomic next-generation sequencing in a patient with peritoneal dialysis-associated peritonitis: a rare case report and literature review. BMC Nephrol.

[11] Nazarenko N, Borkowski P, Berges MM, Varrias D (2023). Lady Windermere syndrome with haemoptysis: suspected pulmonary aspergilloma and MAC pulmonary disease. BMJ Case Rep.

